# Explainable AI Insights Into EEG Classification and Its Alignment to Neural Correlates

**DOI:** 10.1002/hbm.70528

**Published:** 2026-04-26

**Authors:** Hendrik Eilts, Gabriel Ivucic, Niklas Koenen, Marvin N. Wright, Tanja Schultz, Felix Putze

**Affiliations:** ^1^ Cognitive Systems Lab, Universität Bremen Bremen Germany; ^2^ Statistical Methods in Epidemiology, Leibniz Institute for Prevention Research and Epidemiology—BIPS Bremen Germany

**Keywords:** BCI, CRP, EEG, XAI

## Abstract

While deep learning has drastically improved the performance of electroencephalography (EEG) analysis, it remains unclear what these models, such as EEGNet, learn from the data and how their learned features relate to neuroscientific concepts. In this work, we introduce a comprehensive interpretability framework for deep learning models of neural data based on Concept Relevance Propagation (CRP), an extension of layer‐wise relevance propagation that enables the analysis of abstract concepts encoded by individual neurons and filters. We apply CRP to individual filters of convolutional neural networks (EEGNet) trained using leave‐one‐out cross‐validation. To identify common classification strategies across models, we guide the selection of representative data for individual filters using relevance maximization, reduce dimensionality via UMAP, and identify clusters of filters encoding similar concepts through density‐based clustering. To gain insight into the neural correlates of these tasks, we analyze the learned features across multiple data domains without requiring model retraining. We integrate a virtual inspection layer to project explanations into the frequency domain, enabling the simultaneous analysis of spatial, temporal, and spectral aspects using topographic maps, functional grouping, and independent component analysis (ICA). Using three EEG classification tasks—auditory attention, internal/external attention, and motor imagery—we demonstrate that our approach reveals interpretable, task‐relevant neural patterns that generalize across participants. Overall, this framework provides a step toward understanding the models itself and gaining insights into the tasks in terms of neuroscience.

## Key Points

- The generalization of explanation across participants with the goal of finding shared patterns which potentially reveal underlying universal neural processes on three different EEG data sets.
- The identification of useful representations of explanations which we connect to the outcomes of typical methods from the field of neuroscience.

## Graphical abstract

We systematically generate, filter, and cluster explanations of deep learning models of EEG data to identify universal patterns of relevance, which we validate via connections to observations from neuroscience.

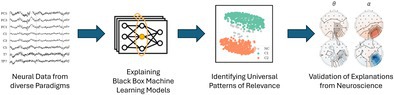

## 1 Introduction

Brain activity data recorded through electroencephalography (EEG) has been processed with methods of machine learning for multiple decades. The most important use case is the development of brain‐computer interfaces (BCIs), which allow communication between a person (e.g., a severely handicapped stroke patient) and a computer through active thought commands or the passive monitoring of user states.

A challenge of applying machine learning to EEG data is that the method is very susceptible to artifacts, for example, caused by movements of the head or eye, which opens the door for a model to rely on spurious relationships between such artifacts and the labels, instead of relying on replicable neural responses. For example, Brunner et al. (Brunner et al. 2010) showed that the popular P300 BCI paradigm, which allegedly captures gaze‐independent attentional responses to visual stimuli, does indeed rely substantially on eye movement patterns. Thus, there is a pressing need to understand the criteria by which a machine learning model decides when classifying neural data.

For traditional BCIs, researchers had the possibility to interpret the weights of their linear models (Haufe et al. 2014) on interpretable features (such as power spectral density in specific frequency bands) which allowed a direct comparison of feature importance to neuroscientific evidence. The recent trend toward using deep neural networks (DNN) for BCI (Craik et al. 2019) results in improved prediction performance. However, this advancement has made model interpretation increasingly difficult due to the sheer complexity and the large number of model parameters (Samek et al. 2021). In this paper, we demonstrate how to bring the necessary interpretability to deep learning models of neural data in a way that is reliable and validated, scaling beyond individual samples, participants, or tasks. The impact of this research is that it provides a conceptual and technical framework applicable to any kind of neuroscientific analysis for bridging the gap between powerful deep learning models and a rigorous process for validation and scientific discovery.

As a consequence of the problem of the interpretability of DNNs, a wide range of techniques has emerged, forming the field of explainable AI (XAI), initially driven by research on computer vision and subsequently adapted to other domains, such as time domain series including EEG data. In general, well‐established model‐agnostic methods such as SHAP (Lundberg and Lee 2017) and LIME (Ribeiro et al. 2016) can be applied to any DNNs, but they are significantly slower than model‐specific methods that can leverage the internal structure in addition to the black‐box prediction function. Moreover, for neural networks, a distinction is made between backpropagation‐based methods like layer‐wise relevance propagation (LRP) and deep learning important features (DeepLIFT) (Montavon et al. 2019; Shrikumar et al. 2017), which follow rule‐based redistribution schemes, and gradient‐based methods like Grad × Input, GradCAM and Integrated Gradients, which rely directly on gradient information (Shrikumar et al. 2016; Selvaraju et al. 2017; Sundararajan et al. 2017). While the latter are less complex and computational demanding, the added flexibility through different redistribution rules and the conversation principle of the former often increase their interpretability.

In the context of neuroscience, Sturm et al. (Sturm et al. 2016) were the first to apply layer‐wise relevance propagation (LRP) to EEG data to investigate neural activity. LRP is a backpropagation‐based method, which utilizes the layer‐wise architecture of a DNN to sequentially redistribute the relevance from the prediction back to the input‐space. This process generates a heatmap in the input‐space, signifying the importance of, for example, pixels or individual time points on a local level (i.e., for a single prediction). The authors analyzed the relevance of single trials in the time domain in the form of scalp topographies, comparing explanations between classes, as well as individual correctly vs. incorrectly classified samples. Similarly, in a study combining LRP explanations and clustering applied to individual time points, Ellis et al. (Ellis et al. 2023) investigated the dynamic functional network connectivity of individuals with schizophrenia. Various studies have adopted backpropagation‐based methods as a means of EEG channel selection. A common approach is to use the frequency domain signal as input (Ellis et al. 2021; Nouri and Tabanfar 2023) or the time‐frequency domain (Zhou et al. 2024), but also by comparing the channel‐wise mean LRP relevance across models (Nagarajan et al. 2023) in the time domain. In addition to the backpropagation‐based method LRP, other approaches such as SHAP explainers have been employed (Putze and Eilts 2023), yielding explanations in the time, frequency and spatial domains on two separately trained CNNs (Raab et al. 2023). Further, perturbation correlations were used to identify neural populations that respond to different properties of the input data, such as amplitude, phase, as well as bimodal neurons (Hammer et al. 2022).

Although many of these methods can be applied to DNNs for BCI, they primarily operate at the level of individual input features, making their interpretations granular yet challenging to contextualize for continuous temporal signals. Even approaches like GradCAM, which involve some degree of aggregation for smoother explanations, do not naturally capture broader temporal dependencies or higher‐level conceptual patterns. Furthermore, they are limited to the signal representation in the input‐domain or to individual predefined features. In terms of trustworthiness, these methods focus on single applications and do not analyze the robustness of explanations across participants and across different training sets. We argue that for a more complete understanding beyond coarse point‐wise feature attributions, encompassing both the model itself and the neural activation patterns associated with a task (neural correlates) contained in the signal, it is necessary to be able to compare the explanations of the input signal in different data domains without having to rely on separately trained models. This would allow analyzing concepts encoded by individual and groups of filters and neurons in terms of different aspects encompassing time, frequency, and spatial domains.

To overcome these limitations, a method that goes beyond point‐wise feature attributions, captures higher‐level concepts encoded within the network, and is flexible for different input domains is required. For these reasons, we chose the method of concept relevance propagation (CRP), which is an extension of LRP (Achtibat et al. 2023). CRP has been applied successfully to several domains, but to the best of our knowledge, never to neural data. This technique allows the analysis of concepts encoded by individual or combinations of filters and neurons within and across layers. In addition, we use a virtual inspection layer (VIL) (Vielhaben et al. 2023), which enables us to project the time series explanations into the frequency domain. We further enhance the CRP output with relevance‐based clustering and a selection of instruments for external validation (e.g., functional grouping, independent component analysis), bringing together traditional neuroscientific approaches with deep learning models.

Given this toolset, we aim to explore (a) the generalization of explanation across participants with the goal of finding shared patterns which potentially reveal underlying neural processes, (b) useful representations of explanations to build a connection to the field of neuroscience.

To this end, we first trained Convolutional Neural Networks (CNNs) in a subject‐independent manner using leave‐one‐out cross‐validation (LOO‐CV) on which we performed our analyses. For a comprehensive analysis of the models, we take into account that individual neurons and filters (also known as “kernels”) may encode separate concepts (Li and Zhang 2023). Moreover, the deeper layers should encode concepts of a higher order, potentially corresponding to distinct neural correlates.

We propose to compute explanations for each individual filter of the last convolutional layer of the network using CRP. The reason we are focusing on the last convolutional layer is that we expect it to contain a representation of abstract neural concepts generalizable across different models and test data sets. Directly comparing the explanations or a subset of samples that lead to a high relevance score is ineffective for this purpose due to the high dimensionality of the data. Instead, we select the samples with the highest relevance (first k samples that make up at least 20% of the total sum of relevance), forward pass them through the model and then use the mean across the resulting activation maps to compute the similarity between filters. For a detailed description and justification of the explanation computation and data selection, we refer to the methods section. Next, we use Uniform Manifold Approximation and Projection (UMAP) with cosine similarity to further reduce the dimensionality, to then employ a clustering algorithm (DBSCAN). Using this approach, we aim to identify clusters of filters that encode similar concepts by looking at the distribution of filters, providing us with a holistic view of the learned representations across the entire network layer through each cluster.

**FIGURE 1:**
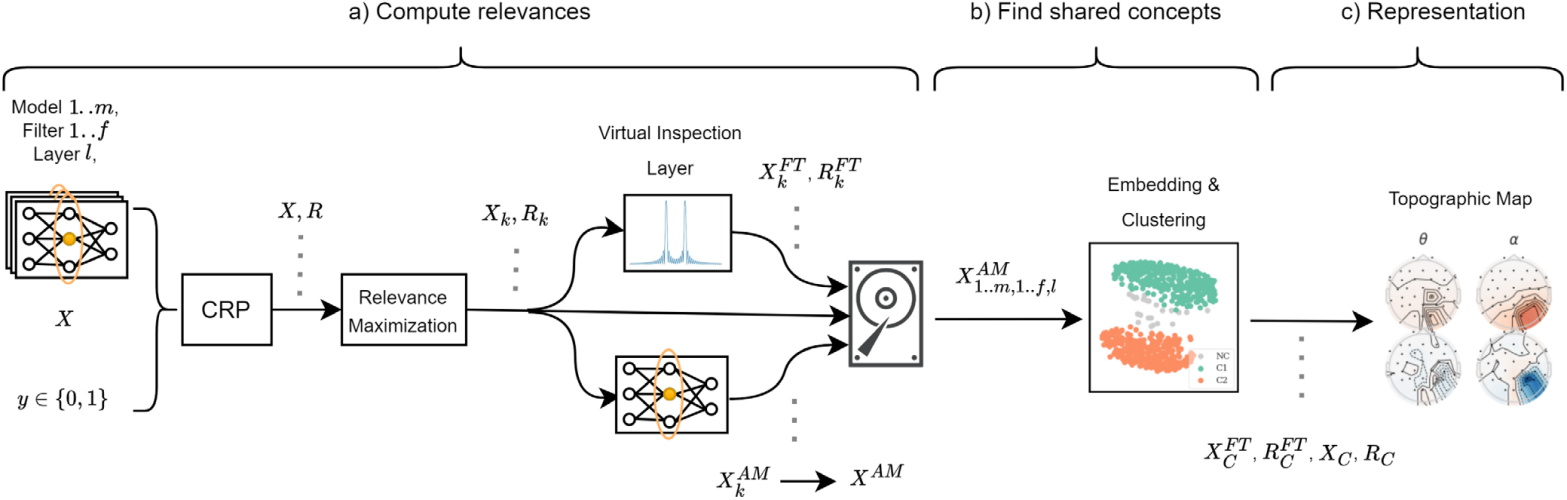
Overview of our pipeline, consisting of three parts: (a) We compute explanations for individual models and filters in a given layer and select high‐scoring samples representing the concept encoded by each filter (relevance maximization). These samples and their explanations are projected into the frequency domain, allowing us to view multiple aspects of the data, and activation maps are computed for the selected samples. (b) The activation maps are used to identify shared concepts across subject‐independent models by applying dimensionality reduction followed by clustering; the resulting clusters represent concepts shared across filters. (c) Finally, we visualize the clusters by aggregating the representative samples for each cluster, using topographic maps to depict both frequency and spatial domains. Here, *X*, *R* denote EEG data and relevance values; subscripts indicate selected samples (*k*) or clusters (*C*), and superscripts indicate activation maps (AM) or Fourier‐transformed data (FT).

The identified clusters are analyzed with established methods of neuroscience: We use topographic maps and functional grouping in the frequency domain. Additionally, we combine the computed relevances with independent component analysis (ICA) to examine the model's use of neural versus non‐neural sources contained in the signal. An overview of this pipeline can be seen in Figure 1.

Furthermore, we analyzed the clusters of similar filters across different model training runs to address our research question of identifying shared patterns that generalize across participants.

We evaluate our approach on three binary EEG classification tasks: internal vs. external attention recognition, left‐hand vs. right‐hand motor imagery and left vs. right auditory attention detection, all of which exhibit well‐established neural correlates. We focus our analysis on the auditory attention detection, a high quality dataset with a large amount of subject data and an expectation of robust lateralized patterns, as well as potential eye‐movement related artifacts for our pipeline to evaluate. We chose the remaining datasets to assess the generalizability of our method with data from which we expected different activation, e.g., in the visual or motor cortex, and different types of artifacts, e.g., induced by body movement.

## 2 Materials and Methods

### 2.1 Data

We tested our approach on three EEG datasets—auditory attention detection, internal vs. external attention detection, and left‐hand vs. right‐hand motor imagery. We selected these three datasets to evaluate our pipeline across a diverse set of binary classification tasks with well‐established neural correlates, two of which exhibit class‐specific lateralization patterns, which we expect to see in the generated explanations. While the binary formulation of the tasks keeps the overall task complexity low, the chosen paradigms span a broad range of neural processes and spectral–spatial patterns. Moreover, the datasets naturally contain artifacts, such as eye movements or muscle activity, enabling an assessment of whether the pipeline captures both neural and non‐neural signals. Finally, the relatively small size of the internal vs. external attention dataset proved valuable for efficient initial validation of the pipeline.

#### 2.1.1 Auditory Attention Detection

The auditory attention dataset was collected by Das et al. (Das et al. 2019). Auditory attention refers to a person listening to a mixture of acoustic sources, but only attending to a single source, such as in a multi‐talker scenario, where the goal is to suppress sounds of the people that the person is not paying attention to (Tzourio et al. 1997). In this dataset, 16 participants listened to Dutch short stories under two audio presentation conditions: dichotic (one story in each ear) and binaural (stories placed 90 degrees apart). Participants focused on the story in one ear while ignoring the other. Each participant completed 8 trials, each lasting 6 min. EEG signals were recorded using the BioSemi ActiveTwo system with 64 channels at a sampling rate of 8196 Hz.

#### 2.1.2 Motor Imagery

The MI dataset by Cho et al. (2017) involved 49 participants imagining moving either their left or right hand, engaging in kinesthetic imagination by touching each finger to the respective thumb. EEG data were collected using the Biosemi ActiveTwo System with 64 electrodes at a sampling rate of 512 Hz. Each participant completed 100 trials, with each trial lasting 3 s.

### 2.2 Internal/External Attention

Vortmann et al. (Vortmann et al. 2019) conducted a study on externally and internally directed attention using an augmented reality task with the Microsoft HoloLens. The dataset, referred to as Dataset IEA, consists of recordings from 15 participants. Each participant completed 72 trials, with the internal attention task lasting 15 s per trial and the external attention task lasting 20 s per trial. EEG data were collected using the g.tec Nautilus mobile 16‐channel system at a sampling rate of 500 Hz.

### 2.3 Preprocessing

For preprocessing, we first filtered out power line noise at 50 Hz and applied a bandpass filter from 1 to 60 Hz to preserve signal integrity while removing unwanted frequencies. Next, we segmented the signal into epochs and resampled them to a frequency of 128 Hz for computational efficiency. We then divided trials into 3‐s windows. To reduce noise, we used a common average reference method, subtracting the average of all channels from each individual channel at every time point. This step helped minimize uncorrelated sources of signal and noise. For standardization, we subtracted the mean and divided by the standard deviation for each channel. To mimic an online recording scenario, we standardized the data in batches of 128 samples during the training and evaluation of the classification model. The other two datasets underwent identical preprocessing steps.

## 3 Classification

### 3.1 Model

For the classification of EEG datasets, we chose EEGNet, as proposed by (Lawhern et al. 2018), due to its strong performance across diverse binary classification tasks. EEGNet is capable of learning temporal and spatial features directly from raw EEG time series, and through its temporal convolutional layers, it can also capture frequency‐specific patterns, which is particularly relevant for our study as we aim to analyze the data across temporal, spatial, and frequency domains. Additionally, the use of depthwise and separable convolutional layers makes the model very parameter‐efficient and provides structural regularization, improving computational efficiency and strong performance even for small to medium‐sized data sets. Furthermore, EEGNet's popularity in EEG research makes it a useful target for analysis, since findings based on this model are likely to generalize to other studies using the same architecture.

We maintained the default hyperparameters as described by the authors, setting $F_{1}$ (number of temporal filters) to 8, $F_{2}$ (number of pointwise filters) to 16, and D (number of spatial filters) to 2. Following the authors' recommendation, we implemented a dropout rate of 0.25 for subject‐independent classification. The Adam optimizer with default parameters, given by Kingma et al. (Kingma and Ba 2017), was utilized to minimize cross‐entropy loss. Each model underwent 500 epochs of training without validation stops, employing a batch size of 128 for training.

The choice of this value is guided by a balance between two conflicting considerations. While a large batch size expedites the training process, the standardization of samples in this work is based on batches. Therefore, a batch size that remains small enough to be viable for an online system was selected.

### 3.2 Leave‐One‐Out Cross‐Validation

We initially trained models in a person‐independent manner using a leave‐one‐out cross‐validation (LOO‐CV) approach. For each LOO‐CV iteration, we assigned a unique random seed to each model. Additionally, we performed another LOO‐CV where each model was assigned the same random seed, enabling us to analyze the effect of non‐determinism on the models, as well as the consistency with which model training learns similar patterns, potentially providing further evidence of the generalization of patterns between models and subjects. This allows us to study the impact of stochastic optimization variance and variations in training data compositions on the presence and stability of generalizable explanations. While it is well‐known that stochastic learning behavior can strongly impact the model weights, this explanation robustness is less studied specifically in EEG.

To ensure the robustness of our results, we repeated the LOO‐CV process 10 times for each condition: that is, models with unique random seeds and models with the same random seed.

## 4 Computing Explanations

In order to understand the concepts a model learned, we can look at individual filters and neurons (units) in a specific layer by choosing representative data using CRP and Relevance Maximization, as shown in Figure 1a. Thus, we iterate over all models, and for each model, we compute explanations and representative data for each unit given by layer l and conditioned on a class label y. But since we are also interested in finding patterns that are shared between participants (RQ (a)), we further compute similarities between the representative data of each unit, allowing us to identify groups of units, that encode similar–but not necessarily identical–concepts, as shown in Figure 1b. Given those groups, we can then visualize the patterns using different representational tools highlighting different aspects, such as topographic maps, Independent Component Analysis (ICA), functional grouping, and so on (RQ (b)).

### 4.1 Data Selection

Since we will use data to represent the patterns learned by filters and neurons, we have to decide whether to use the data a model was trained on or to utilize the test data. The choice determines the potential representation of concepts; in other words, the diversity of the samples limits the representation of concepts. This supports opting for the training set or both training and test data. However, the aim is to identify concepts utilized in person‐independent classification. Therefore, by utilizing the test data, only the concepts essential for that specific individual receive a high relevance score.

As relevance is computed relative to a specific class label, another consideration is to exclusively select samples of the same class. This is because a preliminary analysis revealed that the sample selection can be skewed toward a small pool of highly relevant samples of a single class, regardless of the class conditioned on in CRP. Thus, only samples matching the class of the condition are considered.

### 4.2 Concept Relevance Propagation

For the computation of explanations, we opted for a propagation‐based technique. This approach allows exploring concepts represented by single filters or neurons, facilitating a detailed layer‐by‐layer analysis. For these reasons, we chose CRP, combining local (heatmap) and global explanations to understand where important features occur and what concepts the model encodes (Achtibat et al. 2022).

CRP is a backpropagation‐based attribution method that extends layer‐wise relevance Propagation (LRP). The core idea of LRP is to propagate the prediction score $f\left(x\right)$ backward through the network using local relevance propagation rules []. Given two consecutive layers j and k, the relevance score $R_{k}$ of neuron k is redistributed to neurons j according to

$$R_{j}=\sum_{k}\frac{z_{\mathrm{jk}}}{\sum_{j}z_{\mathrm{jk}}}R_{k},$$

where $z_{\mathrm{jk}}=a_{j}w_{\mathrm{jk}}$ denotes the contribution of neuron j to neuron k, with activation $a_{j}$ and weight $w_{\mathrm{jk}}$.

To support activation functions such as ReLU, the LRP‐0 rule was introduced, which substitutes $z_{\mathrm{jk}}$ for $a_{\mathrm{jk}}$, where $a_{\mathrm{jk}}=\max\left(0,\sum_{0,j}a_{j}w_{\mathrm{jk}}\right)$. In addition, the ϵ‐rule (LRP‐ϵ) introduces a stabilizing term ϵ in the denominator to prevent numerical instabilities, resulting in

$$R_{j}=\sum_{k}\frac{a_{j}w_{\mathrm{jk}}}{\epsilon +\sum_{0,j}a_{j}w_{\mathrm{jk}}}R_{k}$$

Further refinements are provided by specialized propagation rules, such as the LRP‐αβ rule, which treats positive and negative contributions separately:

$$R_{j}=\sum_{k}\alpha \frac{{\left(a_{j}w_{\mathrm{jk}}\right)}^{+}}{\sum_{0,j}{\left(a_{j}w_{\mathrm{jk}}\right)}^{+}}-\beta \frac{{\left(a_{j}w_{\mathrm{jk}}\right)}^{-}}{\sum_{0,j}{\left(a_{j}w_{\mathrm{jk}}\right)}^{-}}R_{k},$$

where ${\left(\cdot \right)}^{+}$ and ${\left(\cdot \right)}^{-}$ denote the positive and negative parts, respectively, and α−β=1. Since the effectiveness of individual propagation rules can vary across layers, it is often beneficial to employ composite rules, in which different LRP rules are applied to different layers of the network, for example using LRP‐0 in upper layers and the LRP‐αβ rule in lower layers.

CRP extends the functionality of LRP by enabling the conditioning on individual or collections of neurons/filters. This is formalized as follows:

$$R_{i\leftarrow j}^{\left(l-1,l\right)}\left(x|\theta \cup \theta_{l}\right)=\frac{z_{\mathrm{ij}}}{z_{j}}\sum_{c_{j}\in \theta_{l}}\delta_{{\mathrm{jc}}_{l}R_{j}^{l}\left(x|\theta \right)},$$

where θ denotes the set of conditioning constraints, selected via the Kronecker delta $\delta_{{\mathrm{jc}}_{j}}$.

For our pipeline, we selected the ϵ‐α2‐β1‐composite, which weighs positive relevance twice as much as negative relevance. This approach focuses on positive evidence while still considering negative evidence.

### 4.3 Relevance Maximization

Given the computed explanations (relevances) R in Figure 1, we perform relevance maximization (Achtibat et al. 2023), that is, we compute the sum of the relevances for each individual sample, which then enables us to select $X_{k}$, the k samples with the highest aggregated relevance.

The choice of k, the number of representative samples of a unit, involves a bias vs. variance trade‐off. Choosing too many samples may include too many secondary characteristics that blur out the actual concept, while choosing too few samples may fail to capture the essence of a concept. Furthermore, the distribution of the relevances of samples follows a somewhat logarithmic curve, which varies from unit to unit. This makes it difficult to determine the best fixed value for k. Instead, k is chosen by selecting the number of samples that make up approximately 20% of the total sum of relevance across all samples for a given unit, leading to values of k ranging between 1 and 10 with a mode of 5.

### 4.4 Unit Representations

The samples $X_{k}$ represent the concept of a unit as a time series signal. However, EEG samples are usually more informative and interpretable in the frequency domain. To address this, a Virtual Inspection Layer (VIL), as proposed by Vielhaben et al. (Vielhaben et al. 2023), is used. This virtual layer is inserted before the original input layer of the classification model and transforms the input signal into the frequency domain ($X_{k}^{\text{FT}}$) and back to the original time signal. This allows relevance to be propagated from the output back to the input layer through the VIL, resulting in relevance scores $R_{k}^{\text{FT}}$ in the frequency domain.

For computing similarity scores between units, a more suitable representation is needed. Similar to (Achtibat et al. 2023), the k selected samples are taken, and a forward pass is performed using the classification model up to the layer in question. The resulting activation maps $X_{k}^{\mathrm{AM}}$ are first aggregated by computing the mean, yielding $X^{\mathrm{AM}}$, and then used as representations to compute the similarity between units in the next step in the pipeline.

Using activation maps is only applicable to convolutional layers. To compute the similarity between neurons in a dense layer, a different representation is required. By passing each sample through the model and recording the scalar activation of each neuron, we obtain a vector of activations for each neuron. This vector can then be used to compute similarities, providing results similar to those from activation maps. However, this method necessitates using all samples (train and test) because using only a subset would render comparing neurons between models impossible. Another option is to use the time series of frequency domain data or the corresponding relevances. However, this approach results in clusters that are highly subject‐specific. Therefore, in this study, we focus on convolutional layers using activation maps.

### 4.5 Clustering

To group units based on their similarity, we take the average activation maps $X^{\mathrm{AM}}$ and project them into a two‐dimensional space using Uniform Manifold Approximation and Projection (UMAP) (McInnes et al. 2020) with cosine similarity, followed by a clustering algorithm. We opted for clustering technique called “Density‐Based Spatial Clustering of Applications with Noise” (DBSCAN) (Ester et al. 1996), due to its flexibility with regard to different cluster shapes, with hyperparameters epsilon = 0.4 and min_samples = 15. An example can be seen in Figure 1b.

This step can potentially be replaced with different clustering approaches. For example, we tested hierarchical clustering and found that it results in very similar groups and patterns.

## 5 Signal Representations

To visualize the patterns encoded by the groups of units found during clustering, we employ several techniques to highlight various aspects of the data associated with each cluster, increasing the interpretability of our results.

### 5.1 Topographic Maps

Firstly, we expect the frequency domain to be more easily interpretable than the time domain. Secondly, we aim to visualize the spatial distribution of the frequency content and its corresponding explanations. Therefore, topographic maps containing the power spectral density (PSD) split into different frequency bands are an efficient way to represent the data. We chose to split the frequency bands according to the widely used ranges: δ (0–4 Hz), θ (4–8 Hz), α (8–12 Hz), β (12–30 Hz), and γ (30–60 Hz), to facilitate comparison with the neuroscientific literature. Furthermore, since the power of EEG signals follows a 1/f relationship with the frequency f, we scaled each band by the average power of each band, but not the corresponding relevance values. For displaying the relevance values in these topographic maps, we only consider the positive values for easier comparison with our other analyses.

## 6 Validation

To further analyze and compare the signal with the literature, we consider two analyses, namely Independent Component Analysis (ICA) and Functional Grouping.

### 6.1 Independent Component Analysis

Since EEG signals are often contaminated with artifacts, such as eye blinks or muscle activity, it is helpful to extract these artifacts and determine to what degree a model relies on these non‐neural signals. We accomplish this by first performing an independent component analysis on the data of each cluster ($X_{C}$) in the time domain, followed by classifying the components using the ICLabel classifier (Pion‐Tonachini et al. 2019), and then projecting the relevance quantity $R_{C}$ of each cluster into the ICA space.

The computation of ICA includes a pre‐whitening step, where all the channels are scaled to unit variance, followed by whitening via Principal Component Analysis (PCA), which transforms the data such that the channels are uncorrelated and have unit variance. The ICA components are calculated using the infomax method, in accordance with the ICLabel classification model (Pion‐Tonachini et al. 2019). The ICLabel classification model classifies ICA components into artifact types such as eye blinks, muscle activity, etc., but also as “brain” if the signal likely originated from neural processes. Given these labeled ICA components, we then project the relevance quantity of a cluster $R_{C}$ into the ICA space by multiplying $R_{C}$ by the precomputed matrices. Aggregating the relevance quantity of each component (taking the absolute value followed by a sum) then yields a scalar relevance value for each labeled component, indicating the importance of this component for the classification.

Additionally, we experimented with bandpass filtering the time series signal prior to computing ICA to analyze artifacts contained in specific frequency ranges. However, this makes the classification results of the ICLabel classifier less reliable, as this model was not trained on such narrow frequency ranges.

### 6.2 Functional Grouping

To facilitate the comparison of signals and explanations between different brain regions, we perform a functional grouping of EEG channels based on regions of interest. For instance, in a Motor Imagery task dataset, we can compare the aggregated signal and relevance between the left and right motor cortices using a bar chart. This approach enables us to quantify differences more easily. However, the results can differ dramatically depending on how the explanations are aggregated. We chose to discard negative relevance prior to taking the average in order to focus solely on positive evidence.

## 7 Explanation Consistency Under Stochastic Variance

To investigate the effect of stochastic variance on the models and the consistency to which model training extracts similar patterns (that CRP can identify) despite different starting conditions, we aim to compare how similar the models are in terms of the patterns they learned. We utilize the average activation maps produced during the computation of explanations. Our approach is as follows:

1. For every combination of two models in a LOO‐CV, we compute the correlation between them. First, we map the filters of model A to those of model B using Cosine Similarity on the average activation maps corresponding to each filter. This step is necessary since the order of filters is arbitrary.
2. We rearrange the average activation maps of model B according to this mapping and flatten these tensors into 1D vectors. Using these 1D vectors, we compute the correlation between models A and B. We repeat this process for all combinations of two models in the LOO‐CV, then average all these correlation values to obtain an overall average correlation for the models in the cross‐validation.
3. We repeat this entire process for each iteration of the LOO‐CV, resulting in 10 different average correlation values.
4. Finally, we perform this entire analysis for two conditions: one with models trained using unique random seeds and the other with models trained using the same random seed.

This method allows us to analyze and compare the consistency of the patterns learned by the models under different random seed conditions.

## 8 Results

As described in detail in Section Materials & Methods, we perform our analysis on three different datasets for which we use the same preprocessing and classification pipeline, using the established EEGNet architecture, for best generalizability of results. For each dataset, we study a binary classification task. Our main example deals with auditory attention detection, i.e., detecting the attended speaker from two human voices played simultaneously from different directions (left and right). The results of the remaining two datasets, internal vs. external attention detection and left‐hand vs. right‐hand motor imagery, are provided in the Appendix. The presentation of the results in the Appendix follows the identical structure as this results section, enabling easy comparisons between data sets.

## 9 Studying Generalizability of Explanations Through Shared Patterns

In order to determine if the EEGNet models trained in the LOO‐CV share common strategies for the classification of left vs. right auditory attention, we clustered the filters of the last convolutional layer using the average activation maps of the most relevant samples, computed using Relevance Maximization for each filter.

**FIGURE 2:**
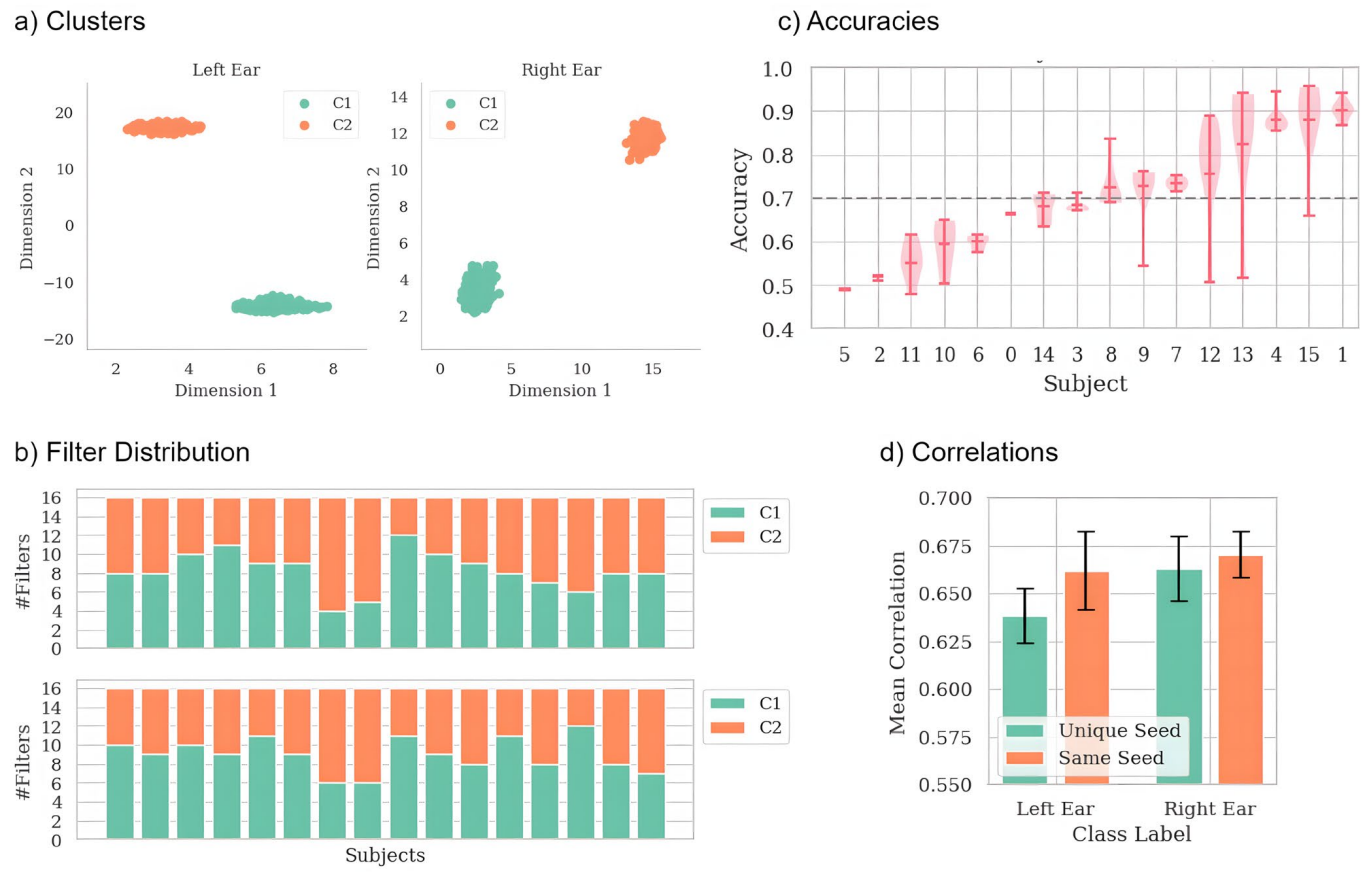
(a) Clustered filters in UMAP space of the last convolutional layer of EEGNet. (b) Shows how each model contributes to each cluster in terms of amount of filters of the convolutional layer we analyze. This layer consists of 16 filters. (c) Accuracy per subject for the leave‐one‐out cross‐validation using EEGNet models on the Auditory Attention dataset. (d) Mean correlation between activation maps of models for different conditions. The error bars show the 95% confidence intervals.

The result for the auditory attention task can be seen in Figure 2a. It is striking how for each class we have a clean separation into two clusters. We observed the same separation into two distinct clusters for our other two datasets. We hypothesize that these clusters must contain filters that share similar classification strategies. However, to determine whether these two groups of patterns are present in every single model of the LOO‐CV and thus generalize across the whole study population, we examine the distribution of filters of each cluster in terms of which model they originate from. This is shown in Figure 2b. It is evident that for each class, both clusters contain filters from every model, indicating that the features encoded by the clusters are present in every model. Furthermore, these features must be present in each subject, as the filters are expressed in terms of activation maps computed from test samples selected through Relevance Maximization for each individual subject. This shows that indeed we find filters that are similar across all folds and thus give generalizable insights into the signal characteristics the model captures.

In addition, we examine how similar the models' learned patterns under stochastic variance. Using average activation maps, we computed correlations between models' activations in LOO‐CV. This process was repeated across 10 iterations, resulting in 10 average correlation values. We analyzed models trained with unique random seeds and those trained with the same random seed to compare pattern consistency. The results are shown in Figure 2d. The mean correlation for the “unique seed” condition is lower than the “same seed” condition for both classes, but more so for the “left ear” class. The error bars indicate the 90% confidence intervals, which overlap between conditions, indicating that the difference is not statistically significant. The fact that the correlations are significantly less than 1.0 in all cases can be attributed to the different subsets of data used in each fold of the LOO‐CV, as well as randomness due to hardware. Thus, while the models tend to learn similar patterns, there appears to be variability, which is slightly amplified when using different random seeds.

## 10 Making Neuroscientific Sense of Relevance Distributions

In this part of the investigation, we relate the findings of the cluster analysis on the generated relevance distributions to the results of applying various established neuroscientific analyses to the data.

### 10.1 Signal Representations

To be able to interpret the signal of the clusters we identified, we consider different signal representations that are more informative than the raw time series signal. For this reason, we consider both the spatial and frequency domain in the form of topographic maps, the Power Spectral Density (PSD) signal distribution along with relevance values.

**FIGURE 3:**
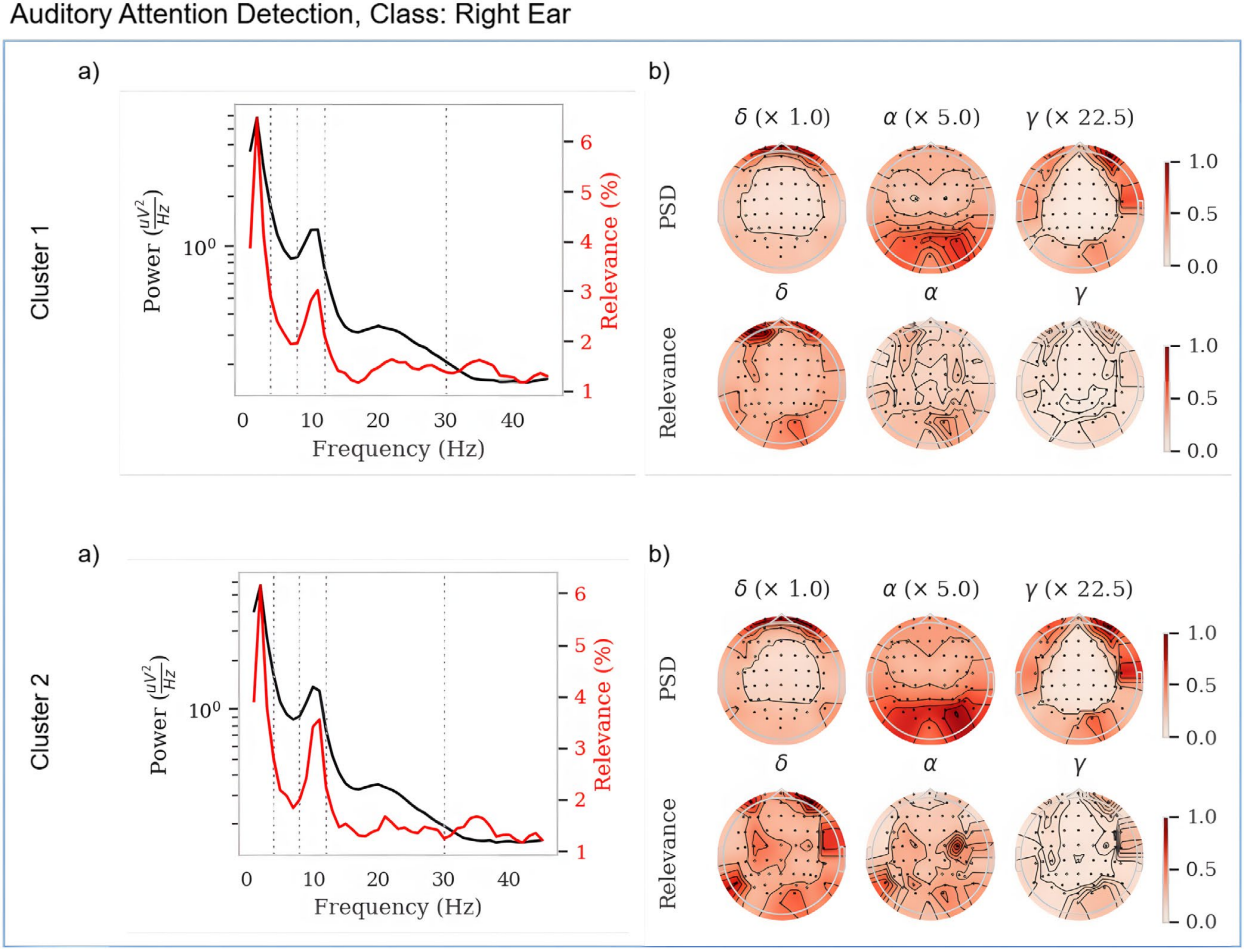
This figure displays results that include power spectral density (PSD), topographic maps. (a) PSD signal up to 45 Hz, along with the percentage of relevance values relative to the total positive relevance. (b) Topographic maps of the PSD signal for selected frequency bands (top row), scaled to half the average frequency of each band. The lower row presents the corresponding relevance values for each frequency band.

Figure 3 provides an overview of our results for the auditory attention detection dataset (AAD). As we only consider binary classification tasks, the results between classes show similar but inverted patterns, which is why we chose to display a single class only. In order to verify our approach, we applied the same analysis on the remaining two datasets, which can be seen in the Appendix Signal Representations.

For the auditory attention detection dataset, we compare both clusters for the class “right ear”. The PSD signals shown in (a) for both clusters have a similar distribution to the previous datasets, with peaks in the δ‐ and α‐bands,^1^ as well as some minor peaks in the β‐ and γ‐bands. Comparing the relevance between clusters, we can see a significant difference in the α peak. Examining the topographic maps in (b) between clusters, we can see that while the PSD signal is very similar, i.e., high power in the frontal lobes, occipital and parietal regions, as well as temporal lobes, the relevance differs substantially. The relevance of cluster 1 is mostly concerned with the left frontal cortex and, to a lesser degree, the right occipital cortex. The positive relevance for cluster 2, however, appears to be strongest in the right temporal cortex, as well as the left parietal and right frontal cortices.

The previous analyses have helped us identify key brain regions involved in the auditory attention task. Next, we employ Independent Component Analysis (ICA) and Functional Grouping to facilitate the validation of our results. The former yields information about the artifacts contained in the signal, while the latter displays the distribution of relevance by grouping brain regions according to their functional roles.

### 10.2 Independent Component Analysis

We performed an ICA analysis on the aggregated data of each cluster to determine if the models focus on artifacts such as eye blinks or muscle activity. For this purpose, we classified the components using the ICLabel classifier (Pion‐Tonachini et al. 2019) into neural and non‐neural sources. Since this is a linear operation, we also projected the relevance scores into the same space, allowing us to provide an aggregated relevance score for each classified component.

**FIGURE 4:**
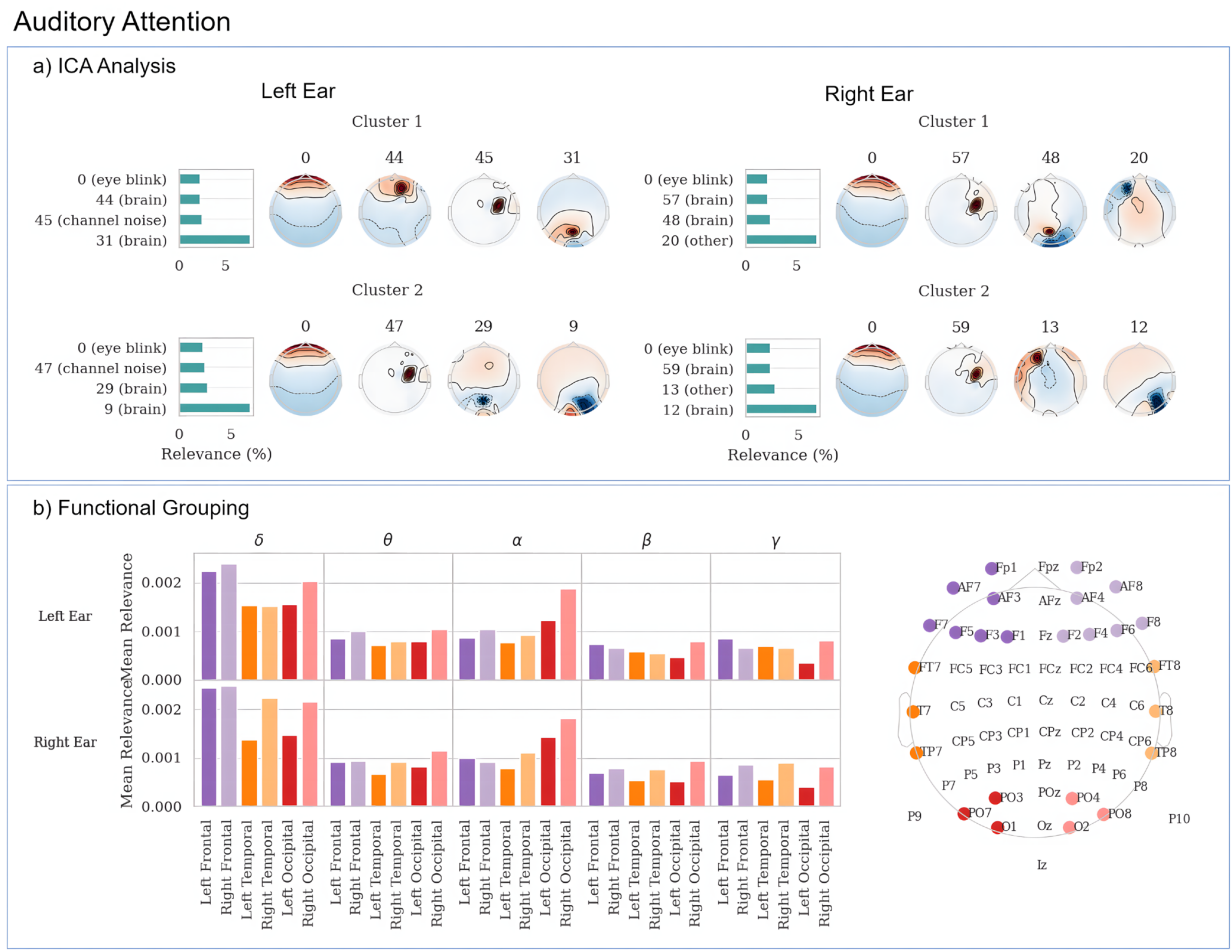
(a) Labeled ICA components with highest relevance, displayed using topographic maps. The number corresponds to the id of the component, while the text in the brackets is the type of artifact the component was classified as. (b) Functional Grouping: Bar charts showing the mean of the absolute values of relevance for brain regions for both classes and different frequency bands.

Figure 4a displays the labeled ICA components with the highest relevance for each class and cluster. For each cluster, the ICA component associated with eye artifacts is among the most relevant, suggesting that eye movements contribute to improving auditory attention classification. The analysis also reveals similar activation patterns to those observed using topographic maps, specifically in the occipital lobe and the right parietal lobe at single electrodes located between the central and right temporal cortex. Components showing single electrode activation in the right central/temporal lobes are present in every case, but are classified as brain signals only once, being identified as channel noise otherwise. Additionally, the clusters contain single electrode activation patterns in the frontal lobes, classified either as brain signals or as “other” (a catch‐all category). The highest‐ranked components are predominantly occipital or right parietal (or both) and are classified as brain signals, with the exception of Cluster 2 of class “Right Ear”, where the dominant component shows a signal of unknown origin from the left frontal cortex. It should be noted that the neural correlates of eye movement can cause neural activation that is strongest in the visual cortex of the occipital lobe, contributing to the observed occipital activations.

### 10.3 Functional Grouping

The result of Functional Grouping is shown in Figure 4b. We chose to compare the left and right frontal cortices, the left and right occipital cortices, as well as the left and right temporal cortices. The topographic map on the right of plot (c) shows the selection of electrodes for each brain region. We included the channels FC5, C5, CP5, FC6, C6, and CP6 for the temporal lobes to include the single electrode activations we identified using the ICA analysis.

Looking at the bar chart, it can be seen that the δ band contains the most relevance for both classes, followed by the α band. The δ band is dominated by the frontal regions as well as the right occipital region, while the α band is dominated by the occipital cortices.

Comparing these relevances between classes reveals several cases of lateralized relevances, where the brain region of one hemisphere exhibits higher relevance than the other hemisphere with a swapped relationship between classes. This is the case for temporal cortices in the δ, θ, β, and γ bands, in the frontal cortices (α, β, γ), but not for the occipital cortices, where the right side contains higher relevances throughout. These lateralized relevances indicate the use of these regions to discriminate between classes, while the high relevance of the right occipital cortex may be related to the overall high power across bands.

## 11 Validation

In the final part of the investigation, we study the validity of the findings by studying the models' classification performance as well as the robustness of explanations against small perturbations in the data splits.

### 11.1 Classification

We trained models in a person‐independent manner using leave‐one‐out cross‐validation, with two conditions: one using unique random seeds and another using the same random seed across models. To ensure robustness, we repeated the LOO‐CV process 10 times for each condition.

Figure 2c illustrates the classification results per subject across the 10 iterations with unique random seeds. The average accuracy over all subjects is 71%, as indicated by the dashed line. We can see that the performance of exactly half of the subjects lies above the average value, and the distribution appears somewhat linear, ranging from 49% to 93%, where values around 50% indicate random guessing due to the binary classification task.

## 12 Discussion

We developed a methodology that facilitates the understanding of concepts learned by deep learning models of EEG in terms of neural correlates, using different representations, that is, different views of the same information. These representations serve as a connecting tool between low‐level explanations and high‐level concepts more appropriate for relating the results to the field of neuroscience. Additionally, our approach helped identify concepts shared between subject‐independent models. This serves as an additional step to discriminate between individual neural activation patterns and those that are fundamental to the given task.

The reported results allow us to draw some important conclusions on applying XAI methods to models of neural data. Through clustering of relevant concepts, we find that the different instances of models learn patterns that generalize across participants and random initialization, offering further evidence beyond above‐chance level accuracies. This is an important observation as it shows that the models do not learn idiosyncratic patterns that depend on specific artifacts or random patterns in the data. Moreover, we demonstrate that the most important concepts are plausible from a neuroscientific perspective.

The analyses of the neural correlates of the clusters we discovered show plausible activation in brain regions associated with each task, respectively. In addition, the relevances are highest in the frequency ranges that, according to the literature, are associated with each task.

Further analysis on the auditory attention dataset also revealed that, while the discovered relevant concepts are mostly of neural origin, they still come from brain regions that in the literature would not be associated with auditory attention processing, but with visual processing in the occipital cortex. Our interpretation is that participants engaged in visual attention naturally as the experiment setup did not control their gaze direction. This observation shows that XAI‐based analysis of statistical models can lead to novel insights into neural processing through a bottom‐up process, complementing a top‐down perspective that is driven by established theory. We propose that such analysis becomes more and more important for complex, less restricted experiments in which not all influencing factors are known prior to the analysis.

Still, our analysis showed a substantial variance among discovered patterns, both across participants and as a result of non‐determinism during the model training. A consequence of this observation is that an aggregated analysis as presented in this work is important to draw conclusions about the models instead of looking at individual samples. This is different from other domains in which the data itself is more accessible to human interpretation, for example with images or text, for which a human observer can relate concept relevances directly to segments or words in the input sample.

Another finding our analysis reveals is that the described approach does not only generalize across participants or data splits, but also across different data sets, as we employ a similar network architecture and hyperparameter choice for classification. The employed data sets differ in terms of electrode distribution, artifact prevalence, underlying cognitive processes, and temporal structure. Future research will extend this scope to other BCI as well as to medical applications.

The presented methods also have a number of limitations. Foremost, the number of concepts the XAI model can identify, in our case locations and frequencies, needs to be specified and implemented by the developers prior to the analysis and the analysis can only provide explanations in terms of these concepts. Furthermore, we only look at a flat clustering of the relevance patterns; a hierarchical clustering could reveal more fine‐granular structures that differentiate the learned patterns further.

Another issue is the low classification performance of some models which could either indicate that the model failed to learn useful patterns due to randomness or due to the exclusion of the test‐subject, or that the test‐subject exhibits activation patterns that are significantly different from the rest. The first case would potentially yield false relationships, while the second case undermines the existence of completely universal activation patterns for a given task.

The pipeline for performing the analysis is available at (Eilts 2025). Other researchers can use it to study their own deep learning models of neural data. Through this process, we show how to establish a new standard for vetting and understanding models of neural data, breaking the double black‐box of hard‐to‐interpret signals and sub‐symbolic model weights open.

Our methodological approach extends prior research by Sturm et al. (2016), focusing more on a global view—specifically, features and concepts that are relevant across models—rather than examining individual trials. This approach is similar to that of Nagarajan et al. (2023), who considered the mean relevance per channel computed via LRP across subject‐independent models and visualized this using topographic maps. Furthermore, we also apply clustering to the explanations like (Ellis et al. 2023), but with a different goal.

A challenge when analyzing different signal domains is the need to train separate models or design specialized networks (Nouri and Tabanfar 2023; Zhou et al. 2024; Raab et al. 2023). We addressed this issue by using the virtual inspection layer (Vielhaben et al. 2023), which not only makes our approach more general but also provides a more comprehensive view of the model itself, enabling us to analyze the features learned by neurons across domains. In future work, it would be valuable to extend this by considering amplitude and phase separately (Hammer et al. 2022), as well as incorporating analyses of the time‐frequency domain (Zhou et al. 2024) and the dynamic network connectivity (Ellis et al. 2023), given an appropriate dataset.

Using our analysis techniques, we identified several brain regions and frequency bands that the EEGNet model relies on for the classification of EEG signals. The literature shows that auditory attention is linked to activity in the auditory cortices of the temporal lobes, but also with brain regions typically associated with visual attention, such as the anterior ocular field and posterior parietal cortex (Smith et al. 2010; Wu et al. 2007), indicating that there is a connection between auditory and visual attention. This is supported by our results. We found high power values in the occipital cortex, especially in the α band, which is consistent with (Wu et al. 2007; Popov et al. 2022). Furthermore, we observed high activations and relevances in the frontal and temporal regions in the time and frequency domains. The involvement of the frontal region is in agreement with (Vandecappelle et al. 2021; Rotaru et al. 2023), who identified the frontal lobe as playing a crucial role in auditory attention independent of ocular‐based artifacts. Our ICA analysis indicates both the activation of the frontal lobes due to eye artifacts and neural origins. The functional grouping analysis further showed evidence of lateralization in the frontal and temporal cortices in the α and β bands, which agrees with (Frey et al. 2014; Gao et al. 2019), as well as the δ band in the frontal and temporal regions. In addition, we also found lateralization in the β and γ bands in the temporal cortices.

## Funding

This work was supported by Deutsche Forschungsgemeinschaft (459360854 and 447089431).

## Ethics Statement

The authors have nothing to report.

## Consent

The authors have nothing to report.

## Figures and Tables

**FIGURE A1:**
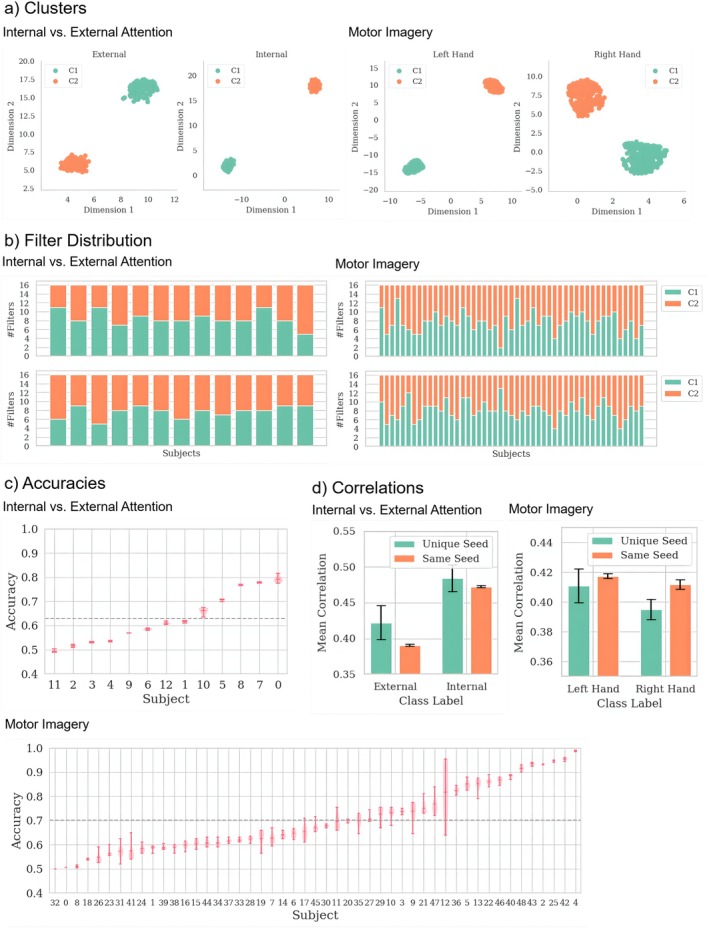
(a) Clustered filters in UMAP space of the last convolutional layer of EEGNet. (b) Shows how each model contributes to each cluster in terms of the amount of filters of the convolutional layer we analyze. This layer consists of 16 filters. (c) Accuracy per subject for the leave‐one‐out cross‐validation using EEGNet models. (d) Mean correlation between activation maps of models for different conditions. The error bars show the 95% confidence intervals.

**FIGURE A2:**
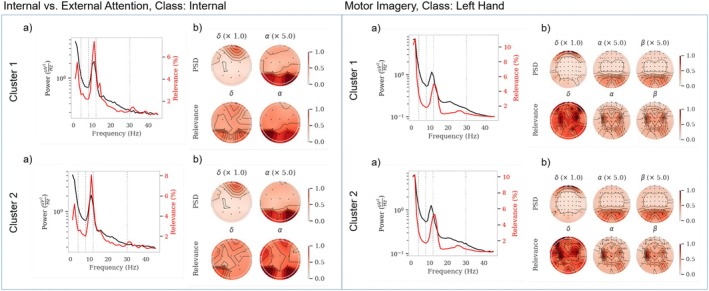
This figure displays results that include power spectral density (PSD), topographic maps for the datasets of internal vs. external attention detection and left‐hand vs. right‐hand motor imagery. (a) PSD signal up to 45 Hz, along with the percentage of relevance values relative to the total positive relevance. (b) Topographic maps of the PSD signal for selected frequency bands (top row), scaled to half the average frequency of each band. The lower row presents the corresponding relevance values for each frequency band.

**FIGURE A3:**
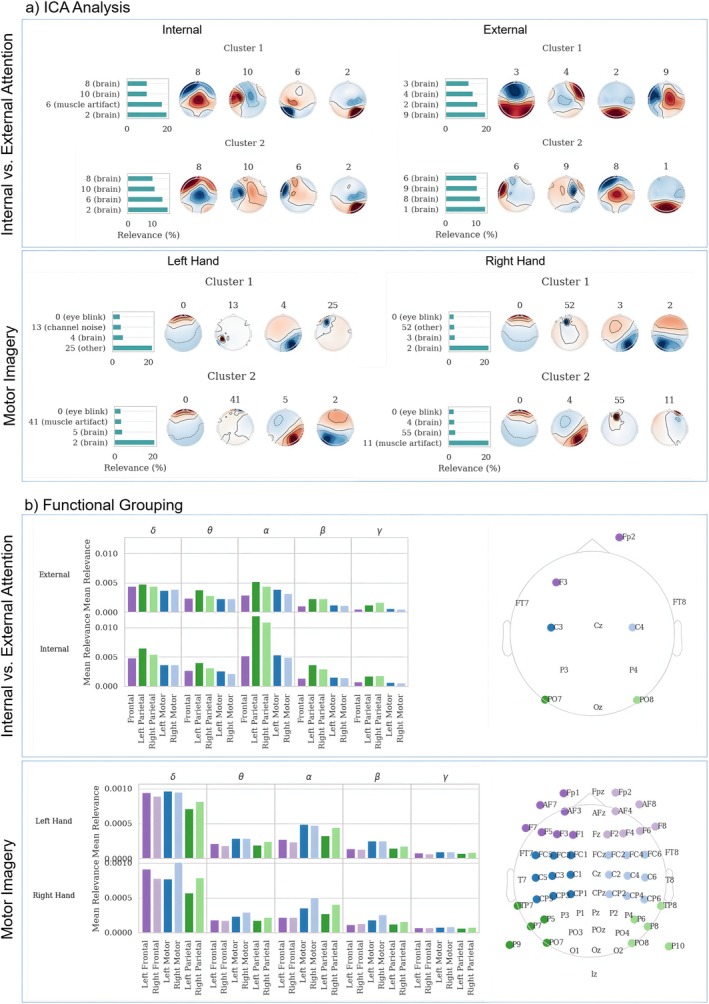
(a) Labeled ICA components with highest relevance, displayed using topographic maps. The number corresponds to the id of the component, while the text in the brackets is the type of artifact the component was classified as. (b) Functional Grouping: Bar charts showing the mean of the absolute values of relevance for brain regions for both classes and different frequency bands.

## Data Availability

Data sharing not applicable to this article as no datasets were generated or analysed during the current study.

## Endnotes

^1^
The frequency bands δ, α, etc. denote frequency ranges which are traditionally associated with certain neural states. For example, strong α (8–13 Hz) synchronization is often associated with a wake resting state.

## Appendix A

This appendix expands on our results by applying the same evaluation to two additional binary classification datasets: internal vs. external attention detection (IEA) and left‐hand vs. right‐hand motor imagery (MI). This analysis is intended to assess the generalizability of our approach. For both datasets, we employed the same preprocessing pipeline, model architecture (EEGNet), and evaluation protocol as used for the AAD dataset. Accordingly, the structure of the analysis mirrors that presented in the main Results section.

### Studying Generalizability of Explanations through Shared Patterns

Figure A1a displays the results of clustering the average activation maps for the IEA and MI datasets. A clear separation into two clusters is observed for each dataset, suggesting the presence of individual, specialized classification patterns. Figure A1b shows the distribution of filters for each model according to their cluster assignment. As in the AAD dataset, the filters within each model are approximately evenly distributed across the two clusters, indicating that both patterns are encoded by each model.

In addition to the presence of shared major classification strategies, we also investigated the effects of stochastic variance, to investigate how consistency of the learned patterns between models despite different starting conditions. To this end we computed correlations between model activations for each individual iteration of the LOO‐CV, trained either with the same random seed or with unique random seeds, in order to assess the effect of randomness on the learned patterns.

The results for the IEA and MI datasets are shown in A1d. Compared to the AAD dataset, correlations are overall lower by approximately 20 percentage points. For the MI dataset, the “unique seed” condition shows slightly lower correlations than the “same seed” condition for both classes. This difference is statistically significant for the Right Hand class, as indicated by the non‐overlapping confidence intervals. In contrast, the results for the IEA dataset show the opposite trend: here, the unique seed condition yields higher correlations for both classes than the same seed condition, and the difference for the External class is statistically significant. The difference between the classes is also striking, suggesting that the External class may be primarily determined by the absence of features associated with the Internal class. Overall, the “unique seed” condition also appears to lead to greater variation in correlation values, resulting in larger error bars.

### Making Neuroscientific Sense of Relevance Distributions Signal Representations

Figure A2 displays the Power Spectral Density (PSD) signal distribution along with relevance values for the IEA and MI datasets. For both datasets we chose a single cluster for which the results appeared most representative in terms of the visual distinctiveness of the patterns in the topographic maps.

For the IEA dataset, (a) shows the PSD signal and its associated relevance for the class “internal”. It is evident that the signal is strongest in the δ and α bands. Observing the topographic maps in (b), we find high power in the frontal lobes, as well as in the occipital and parietal cortices. The relevance values confirm the importance of these regions in both frequency bands.

Looking at the MI dataset for the class “right hand”, we see a similar relevance distribution in (a), but with the peak shifted slightly to the right for the α‐band. This corresponds to the significance of the μ‐rhythm along the motor cortex. This is also evident in (b), where we can clearly identify a peak in relevance on the right motor cortex in the α‐band (which matches the μ‐band in frequency). Furthermore, we also observe high power and relevance in the frontal cortex, especially in the right hemisphere.

### Independent Component Analysis

To determine whether the models trained on the IEA and MI datasets rely on non‐neural signals in the EEG to differentiate between classes, we performed an ICA analysis on the previously identified clusters. The resulting components were classified as neural or non‐neural sources, and their relevance values were examined to assess the contribution of each component to the classification.

Figure A3a depicts these components along with their relevance values. For the IEA dataset and the internal class, the most relevant ICA components tend to be localized over the right parietal cortex in both clusters. Additional notable regions include the left parietal cortex and a left temporal‐to‐frontal region. Activity originating from the left temporal cortex was classified as a muscle artifact, whereas all other components were classified as neural sources. For the external class, none of the components were classified as artifacts. However, the spatial patterns appear more heterogeneous, spanning occipital and parietal as well as central and bilateral frontal regions. For the MI dataset, the results show more focal, point‐like activity patterns, primarily located in the left and right parietal cortices and, notably, also in frontal regions. These frontal components are frequently classified as non‐neural sources, such as eye blinks, muscle activity, or indeterminate (other). This pattern suggests that subjects may have looked toward the left when imagining movement of the left hand and toward the right when imagining movement of the right hand.

### Functional Grouping

Figure A3b depicts the results of Functional Grouping for the IEA and MI datasets. For the IEA dataset, we focus on the frontal, left and right parietal, and left and right motor cortices. The electrode locations corresponding to these regions are illustrated in the topographic map on the right. Comparing mean relevance values between classes, a pronounced difference in the α frequency band is observed for the left and right parietal cortices, regions typically associated with the dorsal and ventral attention networks. In addition, these regions consistently exhibit the highest mean relevance across frequency bands. For the MI dataset, very high mean relevance values are observed in the δ frequency band across all considered regions. Notably, the left and right motor cortices show clear lateralization, most prominently in the α frequency band, which overlaps with the μ frequency range. Furthermore, this lateralization in motor cortex relevance aligns with the task of imagining movement of the left or right hand.

### Validation

#### Classification

Figure A1c shows the per‐subject classification results across 10 iterations with unique random seeds, as described in Section *Classification*. For the IEA and MI datasets, the average accuracies across all subjects are 62% and 70%, respectively. Similar to the AAD dataset, the accuracy distributions appear approximately inear, ranging from 50% to 80% for IEA and from 50% to 99% for MI.
